# Salinity Preference in Hatchery-Reared Juvenile Red Drum

**DOI:** 10.1100/tsw.2002.347

**Published:** 2002-05-15

**Authors:** Daryl C. Parkyn, Debra J. Murie, Edward T. Sherwood

**Affiliations:** Department of Fisheries and Aquatic Sciences, University of Florida, Gainesville, FL 32653;, USA; Florida Marine Research Institute, FWCC, 100 Eighth Avenue S.E., St. Petersburg, FL 33701-5095, USA

**Keywords:** Sciaenops ocellatus, red drum, sciaenidae, salinity preference, twochoice test, stock enhancement

## Abstract

Juvenile red drum (*Sciaenops ocellatus*), reared in either 15- or 30-ppt salinity seawater, were tested to determine whether they develop preference for the salinity of the water in which they were cultured. In a two-choice test, large- and small-sized juvenile red drum chose the raceway that matched the seawater in which they were cultured over the other salinity. Additional large and small fish reared in either 15- or 30-ppt salinity water were also tested following a 4-h acclimation period that simulated the duration of transport time from the hatchery to a release site. These fish also showed preference for their original culture salinity. This observed salinity preference in juvenile red drum has implications with respect to movement or residency of hatchery-reared juvenile red drum out-planted into natural coastal systems.

## INTRODUCTION

Salinity preference, or behavioral selection of a particular concentration of dissolved salts, by an animal has been studied for both fish and invertebrates[1,2,3,4,5,6,7]. Studies have also addressed the issue of salinity as a cue for habitat selection and migration for wild fish[1,3,8]. However, an examination of salinity preferences and whether or not hatchery-reared fish entrain to salinity during culture has not been undertaken. Consequently, the possible implications of these issues on hatchery release programs for estuarine fish have also not been considered.

A recent release–recapture and ultrasonic tracking study in Florida on the post-release survival and movements of hatchery-reared juvenile red drum (*Sciaenops ocellatus*) indicated rapid movement away from the area of release within tidal creeks out into more saline conditions of adjacent coastal waters[9]. While not all hatchery releases of red drum have resulted in rapid emigration from release areas[10], a similar exodus of fish may explain, at least in part, the lack of recaptures previously observed following a release of red drum in Murray Creek, FL[11] and Biscayne Bay, FL[12]. This movement may reflect the previously observed importance of physicochemical variables, such as salinity, temperature, and dissolved oxygen, on growth and recruitment potential or, alternatively, the importance of biological factors such as distribution of predators or size at release[11,12,13,14].

The present study seeks to address whether the salinity of the water during rearing in the hatchery influences preference of salinities of juvenile red drum in a two-choice test. The study examines two major questions: (1) does salinity during culture conditions affect salinity preference, and (2) does short-term adaptation to a different salinity change salinity preference?

## METHODS

### Culture Conditions

Juvenile red drum, reared in brackish water at 28- to 30-ppt salinity, were obtained from the Florida Power Mariculture Facility, Crystal River, FL and transferred to the fish culture facility at the Department of Fisheries and Aquatic Sciences, Gainesville, FL on July 20, 2000. Juvenile red drum were placed into brackish water of either 15 or 30 ppt for a minimum period of 8 weeks prior to testing. Fish were fed pellets (Silvercup Grower®) at a ration of 1–2% body weight every second day. This ration corresponded to the amount of food consumed by the fish in 20 min and was kept low to prevent fouling of the two recirculating seawater systems used for fish culture. Fish were maintained on a 16-h light, 8-h dark photoperiod with water temperature held at 25°C and dissolved oxygen above 6.5 mg $\cdot$ $l^{-1}$. In addition to two salinities, two sizes of red drum were used. Due to fish holding constraints, size-related tests comparing large and small fish were restricted to only fish in the 30-ppt treatment. Mean maximum total lengths (mean ± 1 S.D.) of fish tested from the two salinity conditions were: 182.3 ± 21.3 mm (15-ppt large juveniles); 172.7 ± 14.0 mm (30-ppt large juveniles); 108.1 ± 7.3 mm (30-ppt small juveniles).

### Testing Conditions

Testing was conducted in a flow-through, two-choice raceway (Fig. 1). This test tank was completely surrounded by 1.5-m-high solid, black Coroplast® sheets to serve as a blind, blocking the researcher from the view of the fish at all times. Additionally, observations and scoring of test results were made remotely using a video camera mounted 2 m directly above the tank and connected remotely by cable to a monitor. Fish were first placed in a 20-cm diameter colorless, plastic-mesh cylinder (C) in a release area (RA) to acclimate to the testing conditions for 5 min prior to their release (Fig. 1). A dye test was performed during the initial design of the study to ensure that the mesh permitted free flow of water to the fish during acclimation. The water flow rate on each side of the testing raceway during acclimation and testing sessions was 1 cm $\cdot$ $s^{-1}$ or ~0.05 body lengths $\cdot$ $s^{-1}$ for large fish and ~0.10 body lengths $\cdot$ $s^{-1}$ for small fish. Following acclimation, the cylinder was raised to the ceiling by colorless monofilament line and held at a height 4 m above the surface of the testing tank. A test was considered complete when the fish swam more than half of the way up one of the two raceways (>1 m) (Fig. 1) or when a fish entered a raceway and remained there for a period of 1 min or longer. Fish that did not make a choice within 10 min post-release were excluded from analysis.

Control experiments were first conducted to assess whether a bias existed in the choice of either of the two raceways of the testing tank. Twenty fish from each of the 15- or 30-ppt culture groups were individually placed in the release cylinder (C). Freshly made artificial seawater

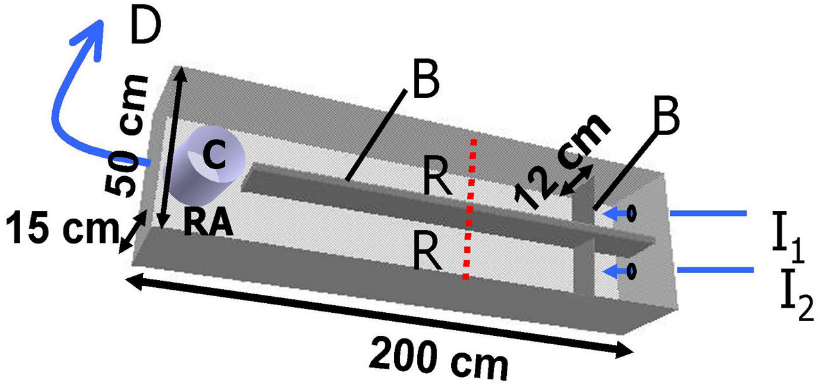

**FIGURE 1**. Top view of the two-channel testing tank. Red dotted line represents position of scoring criterion for tests. B = baffle; C = acclimation cylinder (withdrawn during testing); D = drain; $I_1$ and $I_2$ = water inflows from header tanks; R = raceway; RA = release area.

matching the culture salinity for the control fish was released into both sides of the raceway. Fish were then released and scored as to their choice of one or the other raceway. A two-tailed exact binomial test was used to test that the probability ($p$) that red drum select either raceway is the same (i.e., $H_0$: $p = 0.5$) vs. a bias in selection of raceways ($H_A$: $p \neq 0.5$). Statistical significance for all tests used in this study was defined as $p < 0.05$.

To test whether rearing conditions affect salinity preference, juvenile red drum were placed in the testing tank (Fig. 1) with brackish water of 15 ppt flowing down one raceway and 30 ppt flowing down the other at 1 cm $\cdot$ $s^{-1}$. During testing conditions, salinities of 15 and 30 ppt were randomly assigned to either raceway one or two. A one-tailed exact binomial test was used to test the $H_0$: $p \leq 0.5$, where $p$ is the probability of the fish preferring the raceway with the salinity that was used in its culture. The alternate hypothesis of $H_A$: $p > 0.5$ thereby states that the probability of the fish preferring the raceway with the salinity used in culture was significantly greater than preferring the raceway with the other salinity. These tests were conducted for large fish at both culture salinities (15 and 30 ppt) and for small fish at 30-ppt culture salinity.

As a final experiment, 20 fish cultured in either 15- or 30-ppt salinity were acutely acclimated to 30 and 15 ppt, respectively, over a 4-h period (i.e., the time corresponding to the transport time of fish from the Florida Power Mariculture Center to release areas at the Chassahowitzka National Wildlife Refuge[9]). Therefore, this experiment tested whether short-term acclimation affected salinity preference. A one-tailed exact binomial test was used to test the $H_0$: $p \leq 0.5$, where $p$ is the probability of short-term adapted red drum preferring the raceway with the salinity that was used in its culture. The alternate hypothesis of $H_A$: $p > 0.5$ thereby states that the probability of the short-term adapted red drum preferring the raceway with the salinity used in culture was significantly greater than preferring the raceway with the other salinity.

## RESULTS

Control fish showed no preference for either side of the testing tank regardless of the salinity during culture or size tested (Fig. 2A; 15-ppt large juvenile, $p = 0.82$; 30-ppt large juveniles, $p = 0.82$; 30-ppt small juveniles, $p = 0.82$). In trials of salinity preference, red drum preferred the test channel having the salinity at which they had been cultured significantly more than the test channel with the other salinity (Fig. 2B; one-tailed exact binomial test: 15-ppt large juveniles, $p = 0.006$; 30-ppt large juveniles, $p = 0.001$; 30-ppt small juveniles, $p = 0.021$).

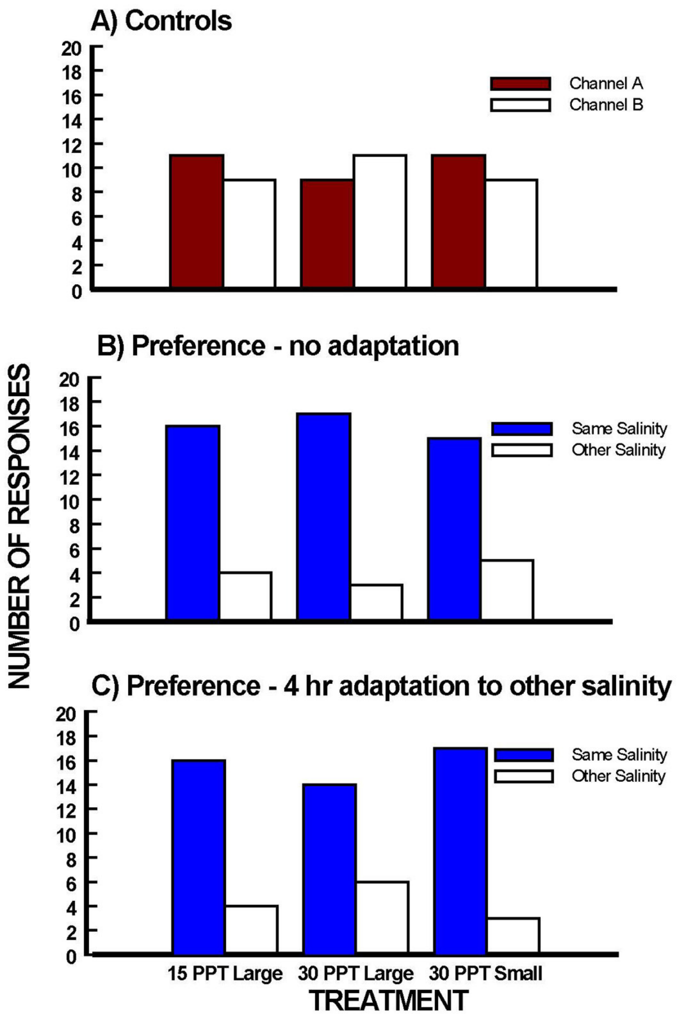

**FIGURE 2**. Total number of positive responses in two-choice tests of juvenile *S. ocellatus*. (A) Control tests to compare raceway selection biases. (B) Preference tests to compare response to culture salinity vs. other salinity. An adaptation time of 5 min was used prior to release from holding area to reduce handling stress. (C) Preference tests to compare response to culture salinity to a second salinity to which red drum had been adapted over the short term. This adaptation time (4 h) corresponded to the time required to capture, transport, and out-plant red drum in a recent study in Florida on red drum hatchery releases[9].

Similarly, in experiments testing short-term salinity acclimation to a different salinity, large red drum juveniles cultured at 15-ppt salinity and both large and small red drum juveniles reared in 30-ppt salinity seawater continued to select the salinity in which they were cultured more frequently than the salinity of their short-term acclimation (Fig. 2C; one-tailed exact binomial test: $p = 0.006$, $p = 0.005$, and $p = 0.001$, respectively).

## DISCUSSION

In the present study, conditions during culture appear to have affected the salinity preference of hatchery-reared juvenile red drum. While the exact duration of this entrainment is currently unknown, we do know that in the 2 months that the fish were held at 15 ppt, we did entrain them to this salinity over their initial culture salinity of 30 ppt. This finding has the potential to be useful in facilitating retention of red drum, in at least the short-term, in the appropriate habitat of release. In particular, this information may aid in better matching of release site characteristics to culture conditions of fish or vice versa. Alternatively, it may also provide clues to improved tracking of the movements of recently released fish.

Red drum is generally considered to be an estuarine-dependent euryhaline species[13,14,15]. However, it has been shown that juvenile red drum physiologically adapt to a wide range of salinities within 24 h of being placed into water with a different salinity[16]. Thus, within the short duration of the present study, the possibility remains that the observed preferences may have a short-term physiological basis. Longer duration studies are needed to test whether salinity preference follows this timeline for adaptation. As an alternative, the observed preference of red drum to a particular salinity may be a function of age-specific imprinting as is observed in salmonids[8,17]. This, however, would seem unlikely because both large- and small-sized red drum responded similarly. This is despite the fact that they were adapted to experimental culture conditions at different times in their development. Regardless of the proximate causes of the observed salinity preference, the implications of these findings to successful out-planting of red drum are potentially profound. One of the primary concerns would be the increased risk of predation due to recently released fish emigrating from the release habitat.

Several studies have suggested the possibility of large-scale emigration from release areas for out-planted juvenile red drum. Willis et al.[11] and Serafy et al.[12] estimated that the disappearance of out-planted red drum exceeded the mortality rate of released fish. Because of the experimental designs used in either study, they were unable to separate mortality from movement. Thus, it was only speculated that emigration might have been a factor[12]. More recently, recaptures at serially displaced stations from release sites in two tidal creeks, in combination with ultrasonic telemetry and release mortality estimates, demonstrated that juvenile red drum released out-of-season were moving several kilometers away from the release area in a matter of a few days[9]. Since wild red drum juveniles can show residency in estuarine creeks for periods of up to 18 months[14], some differences in behavior are clearly present between wild juveniles and these hatchery-released fish. While such emigration by hatchery fish may not necessarily result in increased mortality, it does increase the difficulty of estimating mortality rates over the short term (i.e., actual release mortality) and therefore of determining the longer-term success of a supplemental stocking program.

## ACKNOWLEDGEMENTS

We thank Eric Latimer and David Bruzek of Florida Power and Light for providing the hatchery red drum. We also thank Douglas E. Colle and two anonymous reviewers for their comments on a previous version of the manuscript.
